# A Case of Mistaken Sex

**Published:** 1884-04

**Authors:** Wm. P. McGuire

**Affiliations:** Winchester, Virginia


### A CASE OF MISTAKEN SEX.

WM. P. MCGUIRE, M. D., WINCHESTER, VIRGINIA.

A. B., thirty-five years of age and in good circumstances in life, consulted me on January 12th, 1884, in order to have the sex to which she belonged determined. She was to all outside appearances a fairly formed woman about five feet four inches in height, with long hair curling down her back. Her voice and features were effeminate, and her demeanor was modest. From birth her dress had been that of a woman. All of her associations had been with women, and her business in life that usually followed by that sex. There was no hair upon her face.

I found upon examination that the conformation of her thorax was similar to that of a woman, and that her breasts were developed similarly to those of a young girl. The nipple was erectile. Her arms, hands, and lower limbs were like those of a man. There was a small penis in the natural position about three-quarters of an inch in length, with a well-formed glans and prepuce. It was capable of erection, but had in the glans no aperture. Following from the base of the penis backward was a sulcus about one-half an inch in depth and two and a half inches in length. Lying upon each side of this sulcus, and each inclosed in separate scrotums, were two well-formed and developed testicles, each attached to a moderate sized spermatic cord, the whole conformation resembling the vulva of the female. There was no opening in this sulcus, but just at its posterior termination was an opening one-quarter of an inch in diameter, which was the external opening of the urethra, extending backward and upward into the bladder. No prostate gland was found. She stated that all of her proclivities and desires had been masculine, and admitted that occasionally in her sleep she had pleasurable sensations followed by an ejaculation of a white fluid from the opening of the urethra, which was, of course, an ejaculation of semen. There was no trouble in determining her sex. She was advised to change her dress to that of a man, and to attempt to have by a plastic operation a new urethra made from its termination in the perineum, along the sulcus to the glans penis, in order to effect more convenient urination, as she is now obliged to do so in the sitting posture. —*Medical News.*

